# The effect of stress on the defense systems

**Published:** 2010-02-25

**Authors:** D Dragoş, MD Tănăsescu

**Affiliations:** ‘Carol Davila’ University of Medicine and Pharmacy BucharestRomania

**Keywords:** acute stress, chronic stress, NK cell, lymphocyte, antibody production, vaccination

## Abstract

Acute stress increases resistance to infection. The alteration of this mechanism in chronically stressed people impairs the organism's ability to mount a strong immune response with a resultant increase in morbidity. Acute stress induces a probable sympatho–adrenergically mediated increase in chemotaxis and adhesion molecules expression, thus promoting immune cells migration to sites of infection and/or inflammation, while chronic stress impairs this mechanism. Protracted stressful conditions decrease NK cytotoxic capacity. There is a substance P, which under stressful circumstances mediates the increase in macrophage cytokine production. Acute stress increases T cell mobilization through a beta2–adrenergically mediated process, which is blunted during chronic stress. Psychological stress impairs the immune system's ability to produce antibodies in response to a vaccine, thereby making the organism more vulnerable to infections.

Abbreviations: CRH = corticotrophin–releasing hormone; HPA = hypothalamic–pituitary–adrenocortical; IL = interleukin; NE = norepinephrine; NK = natural killer; SAM = sympathetic–adrenal medullary; S–IgA = secretory immunoglobulin A; SNS = sympathetic nervous system; TGF = transforming growth factor; Th = T helper; TNF = tumor necrosis factor

## Introduction

Nowadays it is a proven fact that psychological stress increases the susceptibility to inflammatory disorders, including those of infectious etiology [1, 2, 3]. Great efforts are directed towards defining the precise pathways mediating this link. The old view was that stressful conditions have a depressing effect on immunity. However, the gathered evidence points towards a more nuanced vision: stress can both increase and decrease the bodily defenses, depending on a diversity of factors such as the duration of the stressful condition or the individual's reaction thereto or perception thereof. Various ways of adapting to the stressful condition may have vastly different repercussions on immunity. 

Confronted with a stressful condition, the organism strives to cope. For millennia, the most often encountered stressors have been the pathogens attempting to penetrate the organism. Therefore, the mechanisms intended to have an adaptive function were evolutionarily designed for the confrontation with these intruders: structures that either bar their entry, or track them down and destroy them. The two main systems at work in this adaptive process are the nervous system (especially the brain) and the immune system. While reacting to a trespasser, there is a continuous interchange of message between these two systems in the attempt to keep the organism in balance and free of infecting organisms. The mechanisms responsible for the mediations of these interactions are both neuroendocrine and autonomic and form the object of a relatively new discipline: psychoneuroimmunology. As the name may suggest, the interactions it deals with are bidirectional: not only do the psychic alterations translate into immune changes, but also products of the immune cells influence neuronal circuits. The major pathways involved in these interactions are the hypothalamic–pituitary–adrenal (HPA) axis and the sympathetic nervous system (SNS).

One of the key mediators is probably corticotrophin–releasing hormone (CRH), which exerts a general immunosuppressive influence by enhancing the release of corticosteroids, catecholamines, and certain opiates through its action on the sympatho–adrenergic system. The release of CRH from the hypothalamus is modulated by behavioral, nervous, and neuroendocrine influences. Stress also modifies the secretion of growth hormone and prolactin, which generally boost the immune system. Although increased early in the stress reaction, in the latter phases these hormones are decreased, being parallel to the similar evolution of the immune response to stress. 

The stimulation of the beta2–adrenoreceptor unleashes the cAMP–protein kinase A pathway, which enhances the humoral immunity, while suppressing the cellular immunity. This change in dominance is exerted by both stimulating the activity of Th–l2 cells and inhibiting the function of antigen–presenting cells and Th–1 cells. We may see this as an attempt to limit the deleterious effects of a non–specific defense reaction, while encouraging a more specific response confined to the aggressor. Therefore, under adrenergic stimulation, the defense reaction shifts from a general systemic inflammatory one to a specific localized long–term protective one. In order to achieve this, the systemic release of proinflammatory cytokines (IL–1, TNF–alpha, and interferon–gamma) is shut down, while that of anti–inflammatory cytokines (IL–10 and TGF–beta) is stimulated. The proinflammatory cytokines (IL–1, TNF–alpha, and primarily IL–8) might still be released in the invaded/ injured region calling for neutrophil accumulation.

The outcome of infectious disease is altered by the chronic exposure to stress [5, 6]. Long–term immune changes are one of the possible pathways through which chronic stress enhances the vulnerability to infectious, neoplastic, and autoimmune disease [7]. The chronic stress–related increased susceptibility to disease is attributable to perturbed homeostatic mechanisms–the term ‘allostatic load’ was created in order to underline the idea that long–lasting adversity is apt to lead the bodily neurochemical activity away from its normal equilibrium (homeostatic) position, with persistent (or even permanent) negative impact on health [8].

One of the many forms of chronic stress is sleep deprivation, which was proven to interfere with the immune processes, altering the function of NK cells, the cytokine production, and the humoral immune responses to vaccination [9, 10, 11].

Altered endocrine mechanisms or maladaptive health practices could serve as a link between stress and impaired immunity [12, 13]. When the environmental challenges exceed the organism's coping ability, major endocrine systems are activated, especially the sympathetic–adrenal medullary (SAM) system (including the SNS and the adrenal medulla) and the HPA system, with a resultant activation of the receptors for cortisol and catecholamines on the leukocytes. This alters cellular trafficking, proliferation and differentiation, and cytokine production [14].

The defense mechanisms encompass several systems, all of which are influenced by stress. These systems may be broadly divided into the innate immune system (including components such as the proteins secreted at mucosal surfaces and the cells involved in killing the bacteria) and the adaptive immune system which has a more specific action and includes antibody production.

### The Effects of Stress on Epithelial Surfaces Protection

The pathogens invasion implies the penetration of epithelial barriers: mucous membranes or skin. The skin's protective mechanisms rely heavily on its thickness (mechanical protection). By contrast mucous membranes cannot be thick, given the secretion and/or absorption processes that are supposed to take place at their surface. Therefore, other protective mechanisms have been developed: certain proteins with more (S–IgA) or less (various secretory proteins) specific protective action (biological protection). All these protective mechanisms, both mechanical and biological, are influenced by stress.

Skin serves as a barrier to prevent the entry of pathogens. It was shown that the recovery of the skin's integrity is delayed in subjects exposed to acute stress [15, 16, 17]. This is in concordance with the protracted healing of wounds after short– and long–term psychological stress [18, 19]. The slow resolution of skin injuries is attributed to stress–induced neural, endocrine and immune alterations: activation of immune and inflammatory processes in the skin, neuropeptide release from the peripheral nerves, and increased systemic glucocorticoid levels [16]. These neural, endocrine and immune changes interfere with processes such as lipid synthesis and cytokine expression, important in the initial phases of wound healing [16, 20]. Research done on animal models proved the involvement of glucocorticoids [21, 22] and of beta–adrenergic agonists in the delayed skin recovery from injury [23].

A wide range of secretory proteins protect the mucosal surfaces: some pertain to the (secretory) adaptive immune system, others to the innate one. The secretory immunoglobulin A (S–IgA) is a member of the adaptive secretory immune system. Its secretion is controlled by neuroendocrine factors and is consequently influenced by stress [2]. The duration of the stressful situation is particularly important: acute stress enhances S–IgA levels, in contrast to prolonged stress, which diminishes S–IgA levels [24].

The secretory proteins with antimicrobial activity are part of the innate immune system and act as a first line, nonspecific protective mechanism. They are produced by the exocrine glands lining the mucous membranes of the digestive, respiratory, and urogenital tracts. Their release is controlled by the neuroendocrine system, which provides the means through which psychosocial stress may exert its influence. It was proven that the secretion of salivary proteins (such as cystatin S, lactoferrin, alpha–amylase, the mucins MUC5B and MUC7) is increased by stressful situations, not only by those requiring an active attitude (with predominantly sympathetic activation), but even more by those inducing a passive one (in which the vagal tone is predominantly increased). The pattern of proteins is also different: only MUC7, lactoferrin, and alpha–amylase are increased when the sympathetic stimulation is dominant, while all the proteins are increased in the vagal tone–increasing situation. This stress–induced change in the pattern and the quantity of salivary proteins may partially explain the way stress alters the susceptibility to infectious diseases [25]. This might have a considerable importance, since circumventing this first line of defense seems to be decisive in initiating an infectious disease. 

The occurrence of an infectious disease can be divided into two distinct phases: the infection (which refers to the microorganisms gaining access into the system), and the disease itself. The increased rate of infectious diseases in stressed individuals seems to be related primarily to an alteration of the first of these two phases: psychosocial stress mainly increases the proportion of infected persons, rather than the progression of infection to clinical disease [26]. The role of the secretory proteins is to prevent the infection; hence they are relevant in the assessment of the stress–related increase in the predisposition to infectious diseases. Protein secretion is primarily controlled by the SNS through beta–adrenergic pathways. 

The effect of usual stressful factors on secretory immunity may be prolonged, lasting considerably more than the actual stressful situations. The exposure to acute stress increases mucin secretion in the digestive and respiratory tracts [27, 28], which should enhance mucosal barrier function. This may appear paradoxical given the increased rate of infection in individuals subjected to acute stress [25]. One explanation may be the adaptive mechanisms developed by the infecting organisms, allowing them to take advantage of the increased protein secretion [29]. One example is Helicobacter pylori; whose ability to bind certain types of mucin proteins enhances the organism's potential to invade the host. It has been proven that under stressful circumstances, the increased secretion of MUC5B promotes the adherence of H. pylori [27]. 

### The Effects of Stress on the Cellular Immunity

Acute stress accelerates both the resolution of an infection [30, 31] and the healing of a wound [32], and may result in an increase in both cellular and humoral immunity [33, 34, 35]. The mediator of these protective actions is, at least partially, the stress hormones-orchestrated mobilization of immune–cells [36, 37]. Leukocyte redistribution is the mechanism designed to provide the adequate number of cells to the sites of invasion/ injury [36]. Chemotaxis and adhesion molecules expression are two critical processes in the recruitment and migration of immune cells to inflammatory sites, promoting the redeployment of leukocytes to places where they are most required in order to fight the potential aggressors.

Immune cells continuously patrol the organism in the quest for intruders. They cover long distances via the circulation, and then exit the blood vessels in various tissues and organs where they exert their function. After a longer or shorter interval, they may reenter the blood vessels and continue to traffic through the body. Psychological stress may alter this pattern of circulation. This may have repercussions on the efficiency of the protective action belonging to the cellular branch of the innate immune system. By activating the adrenergic receptors, the acute stress–induced SAM system stimulation leads to an increase in immunoregulatory cell number and function [38, 39, 40], aimed at increasing the defense ability (it seems sensible that the stress–exposed organism should be more prepared for injury or invasion). However, many researchers have found that the immune response to acute stress in chronically stressed individuals is different from that in healthy persons [41], suggesting a disorder in the sympathetically mediated protective processes.

### The Effects of Stress on the Macrophages

Cellular and humoral immune reactions heavily rely on macrophages, which are sensitive to stress. Stress augments cytokine production, which may explain the increase in acute phase reactants through the induction of their hepatic synthesis by cytokines such as IL–1 and IL–6. Substance P is involved in the stress–induced changes in cytokine levels and in the macrophage responses to stress [42].

### The Effects of Stress on the NK Cells

Acute stress leads to an increase in the number of circulating NK cells [13, 38] by promoting their release from various reservoirs [43]. In young subjects, there may also be an increase in NK cell activity [38]. In stressful circumstances the NK cells lining the walls of the blood vessels leave their waiting position, enter the blood flow, and circulate (through the mediation of various chemoattractants, citokines and ligands) to the various territories were they are needed. The aim of these NK cell mobilizations is to provide protection during acute stress. One of the factors mediating the effects of acute stress on NK cell function is probably the SAM system, through increased levels of NE and/or epinephrine [44]. 

The cell–surface marker characteristic for NK cells is CD56 (besides, NK cells do not express CD3). Functionally, there are at least two subsets of NK cells:

- most of them exert a cytotoxic function and have a low surface density of CD56 (CD56^poor^); these are rich in CD11a adhesion molecules, whose ligands are profusely expressed on the endothelial cells close to the site of an inflammatory reaction; the high density of CD16 on CD56poor NK cells is instrumental in their ability to identify cells coated with antibody inviting NK cells to set free their destructive potentia;
- the remainder, rich in CD56 (CD56^rich^), release cytokines, thereby modulating the function of the immune system is the major NK subtype found in the peripheral lymph nodes. This happens due to a high density of CD62L (L–selectin) adhesion molecules, which promote the migration of lymphocytes into the secondary lymphoid tissues, whose high endothelial venules abundantly express CD62L–ligands.

Common stressors lead to a marked increase in the number of cytotoxic CD56^poor^ NK cells in the blood, which are meant to patrol the organism searching for either intruders or damaged tissue. The SNS activation is a crucial mediator of this process, and the amount of NK cell mobilization parallels the magnitude of other adrenergically mediated physiological changes [45, 46, 47, 48, 49]. It was proven that sympatho–adrenergic tone–increasing conditions (acute stressors, exercise, and catecholamine infusions) selectively increase the number of CD56^poor^ NK cells in the blood [50, 51, 52]. This may be explained by the catecholamine' ability to decrease the CD56 expression on the already circulating NK cells and/or to preferentially mobilize the CD56^rich^ NK cells. The latter hypothesis is better supported by current evidence, pointing to a selective mobilization of NK cells during acute stress, according to differences in surface expression of adhesion molecules [50]. A likely explanation is a higher density and/or sensitivity of adrenergic receptors, which induces a higher responsiveness to catecholamines [51]. Correlatively, during acute stress, the number of circulating CD16 ^rich^ in NK cells increases, thereby promoting the antibody–dependent cytotoxicity [51]. On the other hand, chronic stress results in a decline in NK cytotoxic activity [13]. Therefore, stress seems to have at least some beneficial effects, in the short run, on the immune function and on health in general [36]. It is chronic stress that puts things wrong.

The number of adhesion molecules on the NK cells changes in acutely stressful situations [50, 53, 54, 55]. Acute stressors down–regulate the expression of CD62L on circulating NK cells, while increasing the density of CD11a. Similar changes are induced by strenuous exercise or by pharmacologic stimulation of beta–adrenergic receptors [52]. Beta–blockade has opposing effects [52, 55]. We may conclude that stress–induced changes in adhesion molecule densities on circulating NK cells may be largely attributed to adrenergic hyperactivity.

Conversely, chronic stress reduces the NK cell responsiveness to cytokines [56], with a drop in the NK cell activity, in contrast to the increased activity in normal individuals subjected to an acute stress. NE may be one of the factors mediating this effect, which was shown to reduce NK cytotoxic capacity in vitro [57]. Exposure to chronic stress results in a decreased NK cell ability to kill target cells [13], and diminished response to recombine interferon gamma [58], which may partially explain the chronic stress associated morbidity, as impaired NK function correlates with the development or progression of viral, autoimmune, and neoplastic diseases. However, the immune cell functional capacity recovers relatively quickly: it begins within 1 month and is substantial within 1 to 3 months after the conclusion of the overtaxing situation [59]. Of course, this holds as long as no other major stress supervenes in the next few months.

If exposed to an acute psychological challenge, chronically stressed individuals have a protracted decline in NK cell activity [41] and a more pronounced redistribution of NK cells. Normally, acute stress is associated with a hormonal upsurge in the SAM, HPA and endorphin systems. In individuals previously exposed to chronic stress, these acute stress–induced changes are altered, with an excessive adrenergic response and a blunted beta–endorphinic one [41].

### The Effects of Stress on the T lymphocytes

A brief exposure to stress increases the number of circulating CD8 suppressor/cytotoxic T cells [38]. In healthy individuals acute stress induces not only lymphocytosis, but also a greater increase in CD8(plus)CD62L(minus) T cells compared to CD8(plus)CD62L(plus) T cells. This change may be partially prevented by prior treatment with the nonselective beta–antagonist propranolol, but not by the beta1 selective antagonist metoprolol [60]. In contrast, chronically stressed individuals have a drop in the percentage of circulating CD62L(minus) T lymphocytes [61], which is associated with a decrease in the sensitivity and density of the beta2–adrenergic receptors on lymphocytes [62] and therefore, a drop in the sympatho–adrenergic driven stimulation of immune cells. This may have consequences on the immune cells' readiness to mobilize and migrate in response to acute stressors and implicitly on the organism's ability to defend itself.

Chronic stress decreases the lymphocyte proliferative ability in response to mitogens [63], to lectins, and to T–cell receptor activation [58]. It may also diminish the percentages of total T lymphocytes and of Th lymphocytes, and it may increase antibody titers to Epstein–Barr virus (as a consequence of the cellular immune system's inability to control the latent virus) [64].

### The Effects of Stress on Chemotaxis

Normally, there is an increase in chemotaxis [65] in response to acute stress. However, in chronically stressed persons, the response to typical chemotactic agents is markedly reduced after exposure to an acute stress [66]. In healthy persons, epinephrine has an inhibitory effect on chemotaxis in normal circumstances, but a stimulatory one in response to acute stress, putting the organism in a better position to oppose pathogens. The opposite seems to be the case of individuals exposed to long–lasting stress: usually the chemotactic capacity develops parallel to the level of adrenergic activation, but the relation is reversed in acutely stressful situations. In other words, chemotaxis is active when it is needed in normal persons, in contrast to chronically stressed individuals, where chemotaxis fails when it is most needed and improves otherwise. Therefore, in persons exposed to chronic stress, lymphocytes no longer exert their protective role when necessary; instead they may induce pathological changes (such as atherosclerosis) due to their unduly mobilization in baseline settings. 

Activation of Mac–1 (CD11b/CD18) by chemoattractant agents is involved in leukocyte recruitment to the sites of inflammation. The expression of Mac–1 on lymphocytes is increased during acute psychological stress [65], while the expression of L–selectin (CD62L) is decreased [67, 50]. Correspondingly, the catecholamine levels are positively associated with Mac-1 expression on lymphocytes and negatively associated with L–selectin (CD62L) expression. LFA–1 is a leukocyte integrin, involved in the recruitment to the site of infection, functioning as an adhesion molecule. The infusion of a beta–adrenergic agonist (such as isoproterenol) increases LFA–1 expression on lymphocytes [68], and so does psychological stress [67, 50], while beta–blocking agents (e.g. propranolol) are known to reverse this response [55]. Hence, there is a general agreement that the changes in adhesion molecules expression are due to sympatho–adrenergic activation. However, besides the catecholamines, corticosteroids also seem to be involved in the stress–related changes in leukocyte trafficking [33].

### The Effects of Stress on the Humoral Immunity

Receptors of glucocorticoids (including cortisol) and catecholamines (epinephrine and NE) are found on each of the cell types associated with the primary and secondary antibody response. The increased plasma and tissue concentrations of these hormones during stressful conditions alter the function of these cells, and hence the antibody production. High levels of stress lead to a decrement in the titers of protective antibodies against various pathogens, such as influenza, hepatitis B, and pneumonia [69, 70, 71, 72, 73, 74]. There is convincing evidence about the impact of stress on the secondary antibody response to immunizations, but less so, about the primary one. The feebleness of the evidence regarding the primary response may be due to an inappropriate timing of the assessment, as it is assumed that there are some critical moments when the antibody response might be most vulnerable to stress. There may also be a difference between the effects of long– and short–lasting stress [12]. 

It is believed that there may be a critical period (the 10 days after the vaccine is administered) when stress is more apt to impair the antibody response [75]. Therefore, it seems that the latter stages of the antibody production (which involve cytokine production by activated T cells) are more vulnerable to stress than the earlier stages. This may have therapeutic consequences: psychological interventions are known to increase the humoral immune responses to vaccination [76] and they may be properly scheduled so as to have a maximal impact during the vulnerable period [77]. 

Chronic stress is associated with a diminished antibody response to vaccines [12, 13]. This effect has been most consistently documented in persons caring for a spouse incapacitated with dementia [70, 71, 78, 69, 74, 79]. A bleaker perspective on life might be responsible for this latter effect, as it has been proven that, besides stress, depression is also detrimental to humoral immunity [80]. Nevertheless, one should not forget the immune–modulating effects of genetic factors (such as cytokine gene polymorphisms [80]). 

The effect of acute stress on antibody production seems to be less consistent (the impact on secretory immunoglobulin A production is better proven) and less durable (an association was found only when a tight timing in the assessment of stress and antibody production was observed). Acute stress at the time of antigenic challenge augments the antibody response to both thymus–independent and thymus–dependent vaccines, proving that stress–induced immune enhancement is not dependent on T–cell involvement [81].

Cortisol is one of the pathways through which stress alters the antibody response to a vaccine [82]. Stress–increased cortisol secretion is associated with a feeble antibody response to vaccination [69, 83, 82]. Prolonged stress chronically activates the HPA axis with persistent elevation of systemic glucocorticoids, which may put off balance the Th1/Th2 cytokine network by inhibiting cytokine synthesis. The resultant immune disorders predisposes to a wide range of diseases [84, 85, 86, 87]. Other hormones (such as epinephrine and NE, prolactin, and growth hormone) could also be involved in mediating the link between stress and secondary antibody response [88]. 

Besides the endocrine alterations, lifestyle changes (alteration of alcohol consumption, cigarette smoking, physical activity, and sleep) may act as mediators, linking stress exposure to antibody response [3, 89]. Indeed, smoking, physical inactivity, and sleep deprivation are known to decrease antibody responses to vaccination [90, 9]. Altered sleep patterns also seem to be responsible for the impaired immune response following exposure to stress [91, 92]. Sometimes, a vicious circle may arise, in which sleep deprivation and feelings of stress promote each other [75] to the detriment of humoral immunity.

Psychological interventions aimed at improving stress coping abilities may improve the immune response to vaccines [79].

## Conclusions

Brief acute stressful situations have a stimulating effect on immunity, associated with the general SAM activation, which promotes the redistribution of immune cells from the blood to other organs in the body. However, enduring stress, which is known to result in down–regulation of beta–adrenergic receptors, alters adhesion molecules expression on leukocytes, with a resultant decrease of immune response to acute psychological challenges in chronically stressed persons. In contrast to acute stress, chronic stress impairs NK and T cell function.

Stress modulates the antibody response to vaccinations. Chronic stress–induced by enduring hardship is clearly associated with a decrease in the secondary antibody responses to immunization, especially in older adults.

The immune alterations in stress–generating situations are probably due to hormones (most likely, glucocorticoids and catecholamines) binding to their specific receptors. Behavioral or neurobiological mechanisms might also be operational, as bad life habits (smoking, alcohol consumption, insufficient sleep, poor diet etc.) have well–established repercussions on immunity. 

Proper counseling and psychological support may appease the deleterious effects of chronic stress, helping exposed individuals to preserve the strength of their immune system.

Increasingly, the sympathetic nervous system (SNS) is considered a key mediator, and norepinephrine (NE) is a serious candidate for the role of neurotransmitter/ neuromodulator in the lymphoid organs [4]. There are receptors for catecholamines on the immune cells. The increase in the sympatho–adrenergic tone in response to a stressor results in local and systemic changes is represented by a release of NE from the nervous terminals in the lymphoid organs, in association with increased circulating levels of epinephrine. Subjected to this adrenergic environment, the lymphocytes change their pattern of proliferation, circulation, and cytokine production. 
